# Implementation of a nurse-initiated in-hospital stroke response system: association with early imaging and response standardization in non-specialist wards

**DOI:** 10.3389/fneur.2026.1860811

**Published:** 2026-08-05

**Authors:** Masahiko Ichijo, Kenichi Enatsu, Mitsuki Kyoya, Keitaro Chiba, Takahiro Ogishima, Yohei Sato, Masashi Tamaki, Tomoyuki Kamata

**Affiliations:** 1 Department of Neurology, Musashino Red Cross Hospital, Tokyo, Japan; 2 Department of Neurosurgery, Musashino Red Cross Hospital, Tokyo, Japan

**Keywords:** hospital rapid response team, in-hospital stroke, nurse-initiated, quality improvement, standardization, stroke

## Abstract

In-hospital stroke is associated with delayed evaluation and poorer outcomes than community-onset stroke. We implemented the Musashino In-Hospital Stroke Action Team (MISAT), a nurse-initiated system that summons stroke-specialist nurses when stroke is suspected, and evaluated its association with early imaging and response standardization in non-specialist wards. This single-center retrospective observational study analyzed 80 consecutive MISAT activations for suspected in-hospital stroke in October 2023–December 2025. A pre-implementation cohort served as reference. The primary outcome was time from symptom recognition to brain imaging. Secondary analyses assessed process time metrics and phase-based trends in non-specialist wards. Of 80 MISAT activations, 52 (65.0%) were cerebrovascular events and 28 (35.0%) were stroke mimics. The median [interquartile range] recognition-to-imaging time was significantly shorter post-implementation than in the pre-implementation reference cohort (44.0 [30.0–68.3] min vs. 72.0 [48.0–122.5] min; *p* = 0.002). Recognition-to-activation and activation-to-imaging times were 12.0 [5.0–28.0] min and 28.5 [20.5–38.0] min. In non-specialist wards, recognition-to-activation time improved across phases from 54.0 [21.5–88.0] min to 8.0 [3.0–22.0] min and 10.0 [5.0–17.0] min (Kruskal–Wallis *p* = 0.0039), with reduced variability (Fligner–Killeen *p* = 0.0020). Recognition-to-imaging time also decreased across phases, with reduced variability. The MISAT system facilitated earlier evaluation of suspected in-hospital stroke. In non-specialist wards, improved recognition-to-activation time and reduced variability indicate potential educational and standardization benefits. This nurse-initiated system may be a practical stroke-specific complement to existing emergency response systems.

## Introduction

In-hospital stroke (IHS) is a significant clinical entity that is associated with delayed diagnosis and treatment and with worse outcomes than those in community-onset stroke (COS) (1). Large cohort studies have shown that IHS cases experience delays from symptom recognition to neuroimaging and reperfusion therapy, resulting in less favorable functional outcomes and higher mortality than patients with COS (1, 2).

Several barriers unique to the in-hospital setting may contribute to these delays. In hospitalized patients, neurological symptoms may appear atypical due to postoperative status, infection, sedation, delirium, or metabolic disturbances, thereby complicating stroke recognition itself (3). Furthermore, standard inpatient workflows—including contacting the primary team, waiting for instructions, watchful observation, and arranging transport and imaging—can themselves delay the initial response (4). Similarly, the American Heart Association (AHA) Scientific Statement emphasizes that delays in IHS care can occur at multiple stages, including recognition, activation, imaging, and linkage to treatment, highlighting the need for standardized institutional protocols (4).

Rapid response systems (RRSs) and medical emergency teams (METs) lay the foundation essential for the early recognition and management of deteriorating inpatients (5). However, these systems were originally designed to address respiratory, circulatory, and general clinical deterioration among a broad range of in-hospital emergencies; they do not necessarily ensure stroke-specific symptom assessment, activation criteria, imaging pathways, or rapid linkage to reperfusion therapy (4, 5). Therefore, prompt management of IHS may require a stroke-specific response system that complements existing emergency response structures (4). Improving IHS care requires institutional protocols that incorporate staff education, simple and reproducible symptom assessment tools, clear activation pathways, and rapid access to neuroimaging (4). Established screening tools, such as the Face Arm Speech Test (FAST) and the Emergent Large Vessel Occlusion (ELVO) screen, are critical for initial assessment (6, 7). In particular, establishing a system that directly links bedside nurses—who are often the first to recognize neurological changes in hospitalized patients—to the stroke response team may help reasonably reduce recognition-to-activation delays and promote standardization across the hospital, including non-specialist wards (8, 9). Similarly, the AHA statement emphasizes education, rapid activation, clearly defined roles, and optimized imaging access as best practices for IHS management (4).

Accordingly, we implemented a nurse-initiated IHS response system in which ward nurses directly contact stroke-specialist nurses upon suspecting a stroke, thereby triggering a nurse-led stroke pathway. This study evaluated whether implementing this system was associated with shorter early response times, particularly from symptom recognition to brain imaging. Furthermore, this study assessed its educational and standardization effects in non-specialist wards.

## Methods

### Study design

This single-center, retrospective observational study was conducted at the Musashino Red Cross Hospital, a Japanese tertiary emergency care facility with comprehensive stroke care. Consecutive activations of the Musashino In-Hospital Stroke Action Team (MISAT) for suspected IHS between October 2023 and December 2025 were evaluated.

### Participants

The post-implementation cohort consisted of all consecutive patients who triggered MISAT activation for suspected IHS between October 2023 and December 2025, regardless of the final diagnosis. Thus, both cerebrovascular events and stroke mimics were represented. A pre-implementation reference cohort was established, consisting of patients who underwent brain imaging for suspected IHS before the introduction of the MISAT system. These patients were identified through an electronic medical records search using the keywords “in-hospital ischemic stroke” or “IHS” between January 2020 and September 2023. This keyword-based approach was chosen because its extraction criteria are explicit and reproducible; however, because stroke mimics generally leave no retrievable diagnostic trace before implementation, the pre-implementation cohort consisted entirely of ischemic strokes, without stroke mimics, a difference in diagnostic composition addressed in the Limitations and in a sensitivity analysis. Eligible pre-implementation cases were identified by keyword-based screening and subsequent chart review. These cases were included when brain imaging had been performed for clinically suspected IHS.

### MISAT protocol

**Figure 1:**
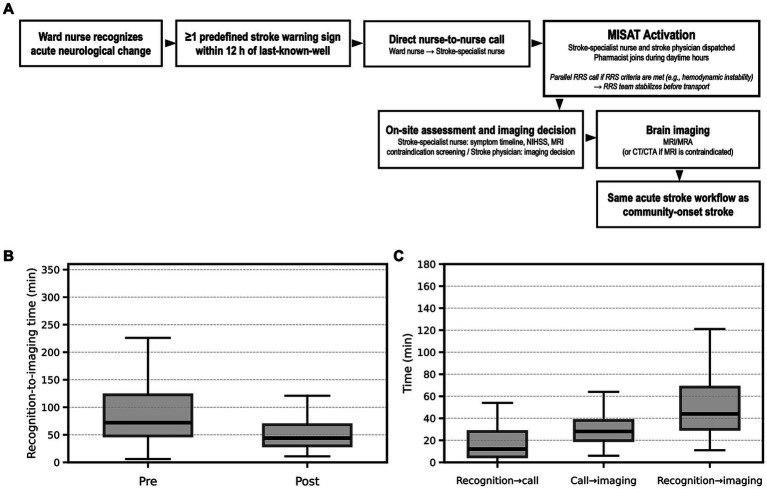
MISAT pathway, pre-implementation versus post-implementation recognition-to-imaging time, and post-implementation process metrics. **(A)** Schematic overview of the nurse-initiated in-hospital stroke response system, Musashino In-Hospital Stroke Action Team (MISAT). Ward nurses recognize acute neurological change and directly contact a stroke-specialist nurse, leading to MISAT activation, on-site assessment, imaging, and subsequent management according to the same acute stroke workflow used for community-onset stroke. **(B)** Distribution of time from symptom recognition to brain imaging in the pre-implementation reference cohort and the post-implementation MISAT cohort. **(C)** Distribution of post-implementation process metrics, including time from symptom recognition to MISAT activation, time from MISAT activation to brain imaging, and total time from symptom recognition to brain imaging.

MISAT is an IHS response system designed for the rapid evaluation of suspected IHS (Figure 1A). Initiated by ward nurses, it directly links bedside recognition of neurological symptoms to the stroke response team. MISAT was activated for hospitalized patients when at least one of six predefined stroke warning signs was present within 12 h of the last-known-well time. These signs included the three FAST items (facial weakness, arm weakness, and speech disturbance) and three ELVO screen cortical signs (aphasia, neglect, and eye deviation). In this study, a stroke-specialist nurse was defined as a nurse routinely engaged in stroke care, with an understanding of hyperacute stroke treatment and the ability to perform a National Institutes of Health Stroke Scale (NIHSS) (10) assessment as part of routine stroke care. Prior to MISAT implementation, nursing staff received annual institutional education on stroke care, including lectures on stroke, NIHSS assessment, and hyperacute reperfusion therapy; thus, ward nurses had an established baseline of stroke-related knowledge. To implement the FAST/ELVO-triggered activation system, several hospital-wide training sessions were conducted for all staff, and MISAT content was subsequently incorporated into periodic educational lectures and reinforced through the hospital intranet. The activation criteria and workflow were documented in the institutional emergency manual, and a MISAT poster together with a ward-specific activation manual was distributed to all wards. When stroke was suspected, ward nurses directly contacted a stroke-specialist nurse via a dedicated phone line. If the patient concurrently met the criteria of the hospital rapid response system (RRS)—for example, in the presence of hemodynamic instability or other acute medical deterioration—an RRS call was issued in parallel, as specified in the MISAT activation flow and communicated to the in-hospital critical care team. In such cases, the RRS team addressed medical instability so that patients were not transported for imaging in an unstable condition. In turn, the stroke-specialist nurse activated the MISAT pathway, prompting the dispatch of both the stroke-specialist nurse and a stroke physician according to the institutional protocol, with pharmacist involvement during daytime hours. On arrival, the stroke-specialist nurse reviewed the symptom timeline with the ward nurse, performed an NIHSS assessment, and screened for contraindications to magnetic resonance imaging (MRI), while the stroke physician determined the imaging strategy and subsequent acute stroke workflow.

### Data collection and definitions

Last-known-well time was defined as the last documented time in the medical records when the patient was confirmed to have no neurological abnormality. Symptom recognition time was defined as the time at which neurological change was first noted, as documented by the nurse. MISAT activation time was defined as the automatically recorded time for the MISAT request. The initial recognizer was defined as the first documented person or staff member to notice the neurological change leading to MISAT activation. Imaging time was defined as the start time of the first brain imaging study (MRI or computed tomography) performed after MISAT activation. Daytime was defined as 8:30 a.m. to 5:00 p.m., and nighttime as all other times. Specialist wards were defined as the stroke care unit and the general ward routinely used for the hospitalization of patients with stroke. All other wards were classified as non-specialist wards. The final clinical and imaging-based diagnoses were used to classify the final diagnoses as cerebrovascular events or stroke mimics. Cerebrovascular events included ischemic stroke, transient ischemic attack, and intracerebral hemorrhage.

### Outcomes

The primary outcome was the time from symptom recognition to brain imaging. Secondary process outcomes included the time from symptom recognition to MISAT activation and the time from MISAT activation to brain imaging. Additional secondary outcomes comprised the final diagnostic categories, including cerebrovascular events and stroke mimics. In non-specialist wards, temporal changes in recognition-to-activation and recognition-to-imaging times were additionally evaluated across three calendar-based phases to assess implementation-related improvement and reduced variability in early response. The three calendar phases were October 2023–June 2024, July 2024–February 2025, and March 2025–December 2025.

### Ethics approval

This study was approved by the institutional review board of Musashino Red Cross Hospital (Approval No. 7035; approved October 2, 2025) and was conducted in accordance with the Declaration of Helsinki. The requirement for individual informed consent was waived because of the retrospective observational study design. Information regarding the study was disclosed using an opt-out approach in accordance with institutional policy.

### Statistical analyses

Continuous variables are expressed as median and interquartile range (IQR), and categorical variables are presented as numbers and percentages. Comparisons between two groups were performed using the Mann–Whitney *U* test for continuous variables, while those among three groups were performed using the Kruskal–Wallis test. Differences in variability across groups were assessed using the Fligner–Killeen test. Significant Kruskal–Wallis tests were followed by *post-hoc* pairwise comparisons using Dunn’s test, with Holm’s adjustment for multiple comparisons. All analyses were performed using JMP software (Version 9.02; SAS Institute Inc., Cary, NC, USA). A two-sided *p*-value < 0.05 was considered statistically significant.

## Results

The post-implementation cohort included 80 consecutive MISAT activations for suspected IHS. Ward nurses were the most frequent initial recognizers of neurological change, accounting for 72/80 MISAT activations (90.0%), whereas physicians and other healthcare staff accounted for 5/80 (6.3%) and 3/80 (3.8%), respectively. Among the 72 activations initially recognized by ward nurses, 59 distinct nurses were involved, each recognizing a maximum of three cases and the majority only one, indicating that recognition was distributed across a broad group of nurses rather than concentrated among a few individuals. The final diagnoses included 52 cerebrovascular events (65.0%) and 28 stroke mimics (35.0%). Cerebrovascular events included ischemic stroke (*n* = 36), transient ischemic attack (*n* = 12), and intracerebral hemorrhage (*n* = 4). The most frequent stroke mimic was seizure, followed by medication- or sedation-related and infection- or sepsis-related conditions (Table 1).

**Table 1:** Baseline characteristics and final diagnoses of the pre- and post-implementation MISAT cohorts.

Variable	Pre-implementation (*n* = 31)	Post-implementation (*n* = 80)
Age and setting		
Age (years), median [IQR]	81 [73–90]	79.0 [70.8–86.5]
Daytime activation, *n* (%)	21 (67.7)	45 (56.2)
Nighttime activation, *n* (%)	10 (32.3)	35 (43.8)
Specialist ward, *n* (%)	4 (12.9)	9 (11.2)
Non-specialist ward, *n* (%)	27 (87.1)	71 (88.8)
Initial recognizer		
Nurse, *n* (%)	24 (77.4)	72 (90.0)
Physician, *n* (%)	3 (9.7)	5 (6.3)
Other healthcare staff, *n* (%)	4 (12.9)	3 (3.8)
Final diagnosis		
Cerebrovascular events, *n* (%)	31 (100)	52 (65.0)
Stroke mimics, *n* (%)	0 (0)	28 (35.0)
Cerebrovascular events		
Ischemic stroke, *n* (%)	31 (100)	36 (45.0)
Transient ischemic attack, *n* (%)	0 (0)	12 (15.0)
Intracerebral hemorrhage, *n* (%)	0 (0)	4 (5.0)
Stroke mimics		
Seizure, *n* (%)	0 (0)	13 (16.2)
Medication–/sedation-related, *n* (%)	0 (0)	6 (7.5)
Infection−/sepsis-related, *n* (%)	0 (0)	4 (5.0)
Syncope/circulatory, *n* (%)	0 (0)	2 (2.5)
Hypoglycemia/metabolic, *n* (%)	0 (0)	1 (1.2)
Other, *n* (%)	0 (0)	2 (2.5)

Continuous variables are expressed as median [IQR], and categorical variables as *n* (%).

Percentages were calculated using the respective cohort totals (pre-implementation, *n* = 31; post-implementation, *n* = 80) as the denominator.

Daytime was defined as 8:30 a.m. to 5:00 p.m. and nighttime as all other times.

Specialist wards were defined as the stroke care unit and C3, a general ward routinely used for hospitalization of patients with stroke. MISAT, Musashino In-Hospital Stroke Action Team; IQR, interquartile range.

The primary outcome, time from symptom recognition to brain imaging, was significantly shorter in the post-implementation cohort than in the pre-implementation reference cohort (44.0 [IQR, 30.0–68.25] min vs. 72.0 [48.0–122.5] min; Mann–Whitney *U* test, *p* = 0.002; Figure 1B).

In a sensitivity analysis restricting the post-implementation cohort to diagnostically comparable subgroups, the reduction in recognition-to-imaging time relative to the pre-implementation cohort remained significant when the post-implementation cohort was restricted to ischemic stroke (*n* = 36; 48.0 [IQR, 32.8–70.0] min; *p* = 0.040) or to all cerebrovascular events (*n* = 52; 48.0 [IQR, 31.8–70.8] min; *p* = 0.019), indicating that the observed improvement was not driven by the inclusion of stroke mimics (Table 2B).

**Table 2:** Process time metrics: primary, phase-based, and exploratory comparisons.

A. Primary outcome between cohort comparison			
Comparison	Pre-implementation reference cohort (*n* = 31)	Post-implementation MISAT cohort (*n* = 80)	*p*-value
Recognition-to-imaging time (min), median [IQR]	72.0 [48.0–122.5]	44.0 [30.0–68.3]	0.002

B. Sensitivity analysis: recognition-to-imaging time (min) by post-implementation diagnostic subgroup			
Comparison	Pre-implementation reference cohort (*n* = 31)	Post-implementation subgroup	*p*-value
Overall MISAT cohort (*n* = 80)	72.0 [48.0–122.5]	44.0 [30.0–68.3]	0.002
Ischemic stroke only (*n* = 36)	72.0 [48.0–122.5]	48.0 [32.8–70.0]	0.040
Cerebrovascular events (ischemic + TIA + ICH) (*n* = 52)	72.0 [48.0–122.5]	48.0 [31.8–70.8]	0.019

C. Overall post-implementation process metrics	
Variable	Overall post-implementation cohort
Recognition-to-activation time (min), median [IQR]	12.0 [5.0–28.0]
Activation-to-imaging time (min), median [IQR]	28.5 [20.5–38.0]
Recognition-to-imaging time (min), median [IQR]	44.0 [30.0–68.3]

D. Phase-based analysis in non-specialist wards				
Variable	Phase 1 (*n* = 11)	Phase 2 (*n* = 25)	Phase 3 (*n* = 35)	*p*-value
Recognition-to-activation time (min), median [IQR]	54.0 [21.5–88.0]	8.0 [3.0–22.0]	10.0 [5.0–17.0]	0.0039
IQR width of recognition-to-activation time, min	66.5	19.0	12.0	0.0020
Recognition-to-imaging time (min), median [IQR]	94.0 [38.0–124.0]	44.0 [28.0–73.0]	44.0 [32.0–50.0]	0.0820
IQR width of recognition-to-imaging time, min	86.0	45.0	18.0	0.0021

E. Exploratory comparison by time of day			
Variable	Daytime (*n* = 45)	Nighttime (*n* = 35)	*p*-value
Recognition-to-activation time (min), median [IQR]	15.0 [3.0–30.0]	10.0 [5.0–20.5]	0.705
Activation-to-imaging time (min), median [IQR]	32.0 [24.0–42.0]	25.0 [16.5–32.0]	0.0056

Continuous variables are expressed as median [IQR]. *p*-values for two-group comparisons were calculated using the Mann–Whitney *U* test. In the sensitivity analysis, the pre-implementation cohort (all ischemic strokes) was compared with progressively diagnosis-matched post-implementation subgroups. TIA, transient ischemic attack; ICH, intracerebral hemorrhage.

*p*-values for three-group comparisons were calculated using the Kruskal–Wallis test. *p*-values for comparisons of variability were calculated using the Fligner–Killeen test.

Phases 1, 2, and 3 were defined as October 2023–June 2024, July 2024–February 2025, and March 2025–December 2025, respectively.

Daytime was defined as 8:30 a.m. to 5:00 p.m. and nighttime as all other times. In two activations, brain imaging had already been performed before MISAT activation was requested; activation-to-imaging time was therefore undefined and these two activations were excluded from all activation-to-imaging analyses (overall *n* = 78; daytime *n* = 43). All other analyses include all 80 activations. MISAT, Musashino In-Hospital Stroke Action Team; IQR, interquartile range.

In the post-implementation cohort, the median time from symptom recognition to MISAT activation was 12.0 [IQR, 5.0–28.0] min, and the median time from MISAT activation to brain imaging was 28.5 [IQR, 20.5–38.0] min. The overall median time from symptom recognition to brain imaging was 44.0 [IQR, 30.0–68.3] min (Figure 1C).

**Figure 2:**
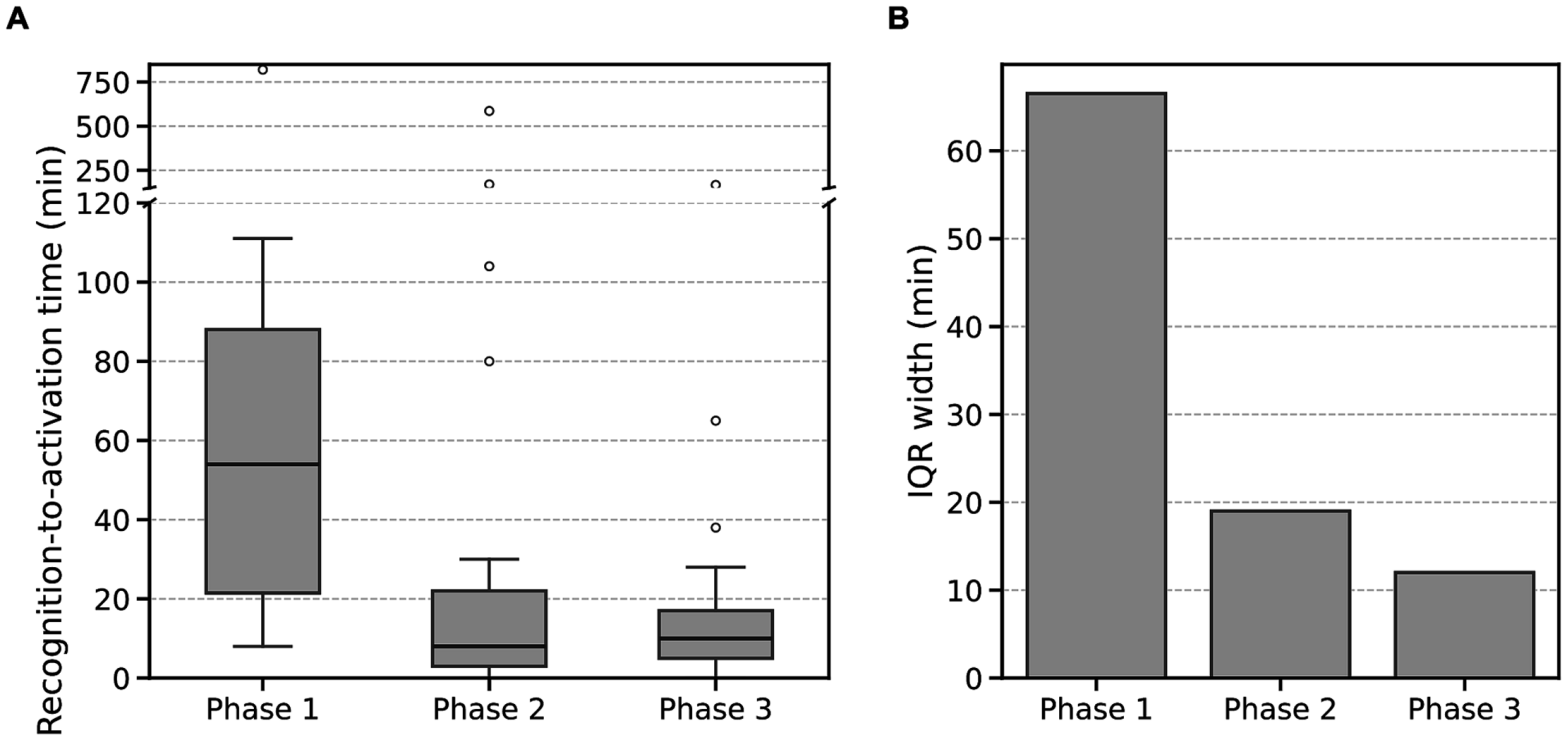
Recognition-to-activation time and variability across three calendar-based phases in non-specialist wards. **(A)** Boxplots showing time from symptom recognition to MISAT activation in non-specialist wards across three calendar-based phases. For visualization, a broken *y*-axis was used to display the main distribution and extreme outliers simultaneously. All observations were retained in the analysis. **(B)** Interquartile ranges (IQRs) of recognition-to-activation time across the same phases, showing reduced variability over time. Phase 1 (*n* = 11), phase 2 (*n* = 25), and phase 3 (*n* = 35) were defined as October 2023 to June 2024, July 2024 to February 2025, and March 2025 to December 2025, respectively.

In non-specialist wards, the median recognition-to-activation time improved across the three calendar-based phases, from 54.0 [IQR, 21.5–88.0] min in phase 1 to 8.0 [IQR, 3.0–22.0] min in phase 2 and 10.0 [IQR, 5.0–17.0] min in phase 3 (Kruskal–Wallis, *p* = 0.0039; Figure 2). Variability in recognition-to-activation time also decreased across phases, as reflected by the narrowing of the IQR (Fligner–Killeen, *p* = 0.0020; Figure 2). In *post-hoc* pairwise comparisons, recognition-to-activation time in phase 1 differed significantly from that in phase 2 (*p* = 0.0054) and phase 3 (*p* = 0.0054), whereas phases 2 and 3 did not differ (*p* = 0.905; Dunn’s test with Holm’s adjustment). Recognition-to-imaging time in non-specialist wards decreased from 94.0 [IQR, 38.0–124.0] min in phase 1 to 44.0 [IQR, 28.0–73.0] min in phase 2 and 44.0 [IQR, 32.0–50.0] min in phase 3, although the difference in median values was not significant (Kruskal–Wallis, *p* = 0.0820). Variability, however, decreased significantly across phases (Fligner–Killeen, *p* = 0.0021).

In exploratory analyses, the activation-to-imaging time was shorter during the nighttime than during the daytime, whereas no significant difference was observed in recognition-to-activation time. Detailed exploratory comparisons are summarized in Table 2.

## Discussion

In this single-center retrospective study, the implementation of a nurse-initiated IHS response system was associated with a shorter time from symptom recognition to brain imaging than in the pre-implementation reference cohort. This finding is clinically significant because IHS has consistently been linked to delayed evaluation and poorer outcomes compared with COS (1, 2). Furthermore, in non-specialist wards, recognition-to-activation time improved across calendar-based phases, and variability narrowed over time, suggesting potential educational and standardization benefits of the system (4).

The improvement in recognition-to-activation time is important because delays in intervention for IHS often arise before patients enter the imaging and treatment pathway (4). Identifying stroke in hospitalized patients is frequently complicated by postoperative status, infection, sedation, delirium, metabolic disturbances, and the higher prevalence of stroke mimics (3). Furthermore, routine inpatient workflows—such as contacting the primary team, waiting for instructions, and arranging transport—may further delay activation of the response system and subsequent evaluation (4). Taken together, our findings suggest that one modifiable target for implementation may be the interval between bedside recognition and formal stroke team activation, especially in non-specialist wards.

In our cohort, ward nurses were the most frequent initial recognizers of neurological changes, accounting for 90.0% of MISAT activations. This finding underscores the rationale for a nurse-initiated activation pathway. In this context, a system that enables ward nurses to directly contact stroke-specialist nurses may minimize delays inherent to hierarchical communication and may accelerate entry into the stroke workflow (9). Although our study did not directly compare different organizational models of IHS response, the reduced recognition-to-activation time supports the practical value of allowing bedside nurses to trigger stroke evaluation without waiting for intermediate hierarchical communication. Our results are consistent with previously reported nurse-driven in-hospital stroke pathways. Yang et al. (11) reported an interdisciplinary, nurse-driven in-hospital code stroke protocol that reduced symptom-to-imaging time from a median of 69 to 37 min, and Siaron et al. (12) described a rapid response team–driven model in which nurse-initiated activations achieved faster code-to-imaging times. In contrast to these models, which integrate stroke response within an existing rapid response team, MISAT establishes a direct nurse-to-nurse activation route to stroke-specialist nurses and, to our knowledge, represents one of the first nurse-initiated in-hospital stroke pathways reported from Japan. Moreover, our phase-based analysis showed progressive reductions in both recognition-to-activation time and its variability in non-specialist wards, which has been less emphasized in prior reports. Future multicenter studies are needed to establish whether a nurse-initiated activation model offers distinct advantages over other stroke team-based activation systems. This approach may be particularly beneficial in non-specialist wards, where stroke presentations are less common, and a simple, structured escalation route could enhance response reliability (4).

A substantial proportion of MISAT activations were ultimately classified as stroke mimics. Because diagnostic uncertainty is inherent to IHS evaluation, this finding should not necessarily be interpreted as inappropriate overactivation, where neurological symptoms are often obscured by comorbid illness, treatment effects, and nonvascular conditions (3). In such settings, prioritizing system sensitivity is clinically justified, especially when the primary goal is to prevent missing time-sensitive cerebrovascular events (4). Consequently, our results suggest that the inclusion of stroke mimics may represent an acceptable tradeoff for earlier recognition and activation, provided that the downstream workflow remains efficient.

These results should not be interpreted as a replacement for existing RRSs or METs. Instead, they highlight the utility of a dedicated stroke-specific in-hospital pathway that functions alongside broader emergency response structures (4). RRSs and METs are designed to identify and respond to a wide range of clinical deterioration symptoms across diverse conditions, whereas acute stroke requires additional disease-specific processes, including focused neurological assessment, explicit activation criteria, and rapid linkage to neuroimaging and reperfusion workflows (4, 5). In this regard, MISAT functions as a practical hospital-based stroke pathway embedded within existing emergency care systems, with particular relevance for institutions seeking to improve recognition and escalation outside dedicated stroke units.

This study has several limitations. First, it was a single-center retrospective observational study, which limits the generalizability of findings and precludes causal inference. Second, the pre-implementation reference cohort was constructed via an electronic medical records search using predefined keywords. Some patients with suspected IHS may not have been identified if the relevant terms were not documented. Moreover, because stroke mimics generally leave no retrievable diagnostic trace before implementation, the pre-implementation cohort consisted entirely of ischemic strokes, whereas the post-implementation cohort included a substantial proportion of stroke mimics. To address this difference in diagnostic composition, we performed a sensitivity analysis restricting the post-implementation cohort to diagnostically comparable subgroups, in which the reduction in recognition-to-imaging time remained significant (Table 2B). Third, symptom recognition time was retrospectively determined from clinical documentation, which may have been recorded inconsistently, particularly during the earlier implementation phase in non-specialist wards. Therefore, the observed phase-based reduction in recognition-to-activation time may reflect not only genuine improvements in workflow but also greater documentation consistency, repeated education, feedback, and increased staff familiarity over time. Although a learning effect cannot be excluded, recognition was distributed across a broad group of nurses (59 individuals), with no more than three activations attributable to any single nurse, arguing against the possibility that repeated recognition by a limited pool of nurses independently drove the reduction in recognition time. To the extent that such an effect occurred, it is consistent with, rather than distinct from, the educational and standardization benefit that the system is intended to confer.

Furthermore, the pre-implementation period overlapped with the COVID-19 pandemic, which has been associated with disruption of stroke care pathways. However, the reported impact of the pandemic has predominantly affected the prehospital interval of community-onset stroke, whereas its effect on purely in-hospital processes such as door-to-imaging has generally been modest (on the order of a few to approximately 10 min), a magnitude unlikely to account for the difference in recognition-to-imaging time observed here. Moreover, the phase-based improvements in non-specialist wards occurred entirely within the post-implementation period. While a before–after design cannot fully exclude pandemic-related temporal confounding, these considerations suggest that the observed improvements are not solely attributable to resolution of the pandemic. In the present study, we primarily focused on early process metrics and did not evaluate functional outcomes, mortality, or reperfusion-related clinical endpoints. Future studies should examine whether earlier recognition and imaging facilitated through systems such as MISAT lead to improved treatment rates and clinical outcomes, including in patients undergoing mechanical thrombectomy.

In conclusion, a nurse-initiated IHS response system can expedite the evaluation of suspected IHS. The reduction in recognition-to-activation time and variability over time in non-specialist wards indicates potential educational and standardization benefits. Ultimately, this approach offers a practical stroke-specific pathway to complement existing hospital emergency response systems.

## Data Availability

The raw data supporting the conclusions of this article will be made available by the authors, without undue reservation.
